# Case report: Left gaze and facial nerve palsies after ventral intermediate thalamic nucleus deep brain stimulation implantation

**DOI:** 10.3389/fneur.2023.1130087

**Published:** 2023-03-02

**Authors:** Victoria Cegielski, Sean Gratton

**Affiliations:** ^1^Department of Neurology, School of Medicine, University of Missouri–Kansas City, Kansas City, MO, United States; ^2^Department of Ophthalmology, School of Medicine, University of Missouri-Kansas City, Kansas City, MO, United States

**Keywords:** neuro-ophthalmology, gaze palsy, facial nerve palsy, neurology, ophthalmology

## Abstract

Deep brain stimulation (DBS) to the ventral intermediate nucleus (VIM) of the thalamus has become a common procedure for some refractory, medication-resistant movement disorders like essential tremors. The most common adverse effects from this surgery include dysarthria and gait disturbances. This case report details a left gaze and ipsilateral facial nerve palsy following overshot cannula insertion into the pons during a VIM DBS procedure. Initial patient presentation after surgery revealed significant impairment of horizontal gaze to the left. This improved during follow-up visits and after the recession of the bilateral medial recti. When considering complications of the VIM DBS procedure, surgeons should be aware of the risks of cannula overshot given the anatomic proximity between the thalamus and brainstem. Furthermore, patients should be aware of this risk when making their surgical decision. All patients who undergo VIM DBS should be assessed for cranial nerve deficits after placement.

## Introduction

Advancement of deep brain stimulation (DBS) over the past few decades has progressed the treatment and knowledge of various medical conditions. DBS is a neurosurgical procedure that involves implanting electrodes in specific areas of the brain to intercede misconducting circuits (1). In the field of neurology, DBS has become one of the treatments of choice for severe motor disorders, including Parkinson's Disease, dystonia, and tremor (2). Essential tremor is the most common tremor disorder, characterized by its worsening with action, including postural and/or kinetic exacerbation. Most commonly seen in the upper limbs, it is often unresponsive to first-line medications, including propranolol, and primidone (3). Thalamotomy of the ventral intermediate nucleus (VIM) was previously used to treat disabling, treatment-resistant tremors. With advancements in technology, DBS to the VIM has replaced thalamotomy as the procedure of choice (4). Dysarthria and gait disturbances are the most reported adverse effects of VIM DBS (5, 6). This study presents in detail a case of VIM DBS that resulted in left gaze and facial palsy complications.

## Case description

### Movement disorder clinic

A 69-year-old right-handed woman was being followed for a 30-year history of essential tremors. This was a bilateral upper extremity postural and kinetic tremor, slightly worse on the right side, that had worsened over the past 5 years. Baseline tremor without primidone was more than 50% worse compared to primidone 50 mg two times daily. She had also tried propranolol in the past, but this was stopped due to low blood pressure. She complained that her tremor interfered with many daily activities and hence she wanted to proceed with DBS. A neurological examination showed to be negative for any focal findings outside of tremor. Neuropsychiatric testing revealed intact cognitive functioning. After a discussion with the patient, a referral to neurosurgery was placed for DBS of bilateral VIM.

### VIM DBS procedure

The scheduled procedure was a bilateral craniotomy for the placement of subcortical leads with the VIM nucleus as the target for the implant.

**Figure 1:**
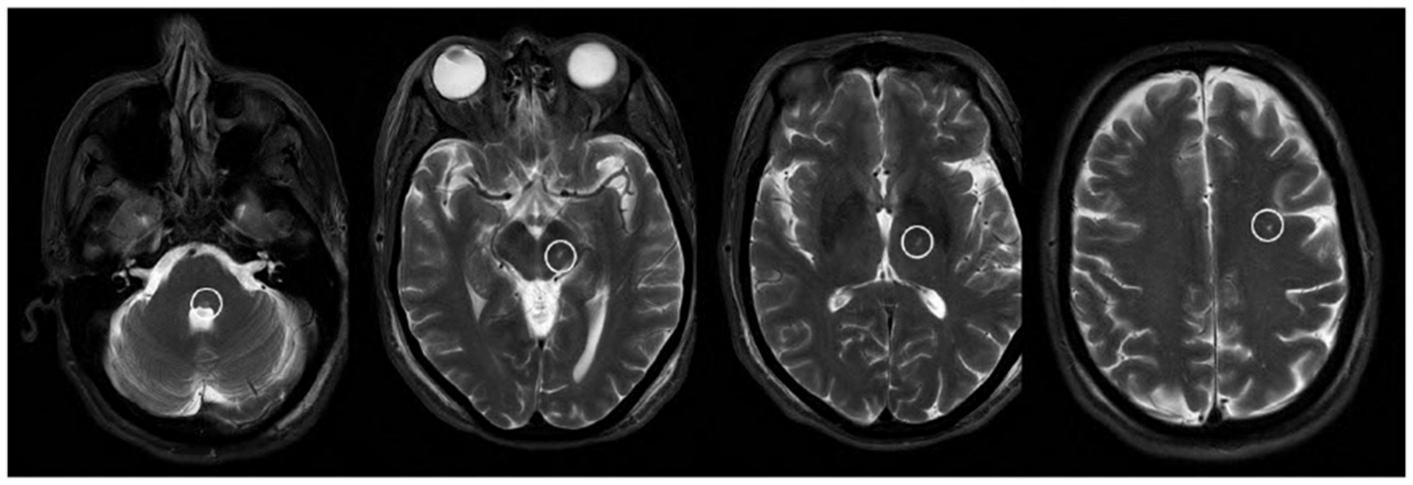
T2-weighted MR images showing the cannula tract as a T2 hyperintensity extending from the dorsal pons through the midbrain, thalamus, and subcortical white matter.

The patient was brought to the operating room and positioned supine on the table. An initial stereotactic computed tomography (CT) scan was obtained. The stereotactic frame CT was then fused to the patient's preoperative CT and magnetic resonance imaging (MRI) imaging to plan DBS trajectories. The patient's head was flexed and fixed to the robotic stereotactic assistance (ROSA) robot with planned incisions. After the left incision was opened, a burr hole was made and widened. The outer table was drilled down, and the hole was fixed over with titanium screws. With an attached Stardrive, the ROSA robot was driven to the left trajectory. The cannula with target depth was placed in the central channel. At this point, an intraoperative CT was fused to preoperative imaging and showed a depth >5 cm past the target. Due to concern for possible neurological and/or vascular injury, the cannula was immediately withdrawn, and the case was aborted. After wound closure and the patient's awakening, the patient was transferred to CT. Though no intracranial hemorrhage was noted, when the patient was moved to the Neuro ICU, she was noted to have left upper and lower facial weakness and limited abduction of the left eye. MRI of the brain showed the cannula tract through the brain parenchyma, extending from the left calvaria to the left dorsal pons (Figure 1).

### Post-operative hospital course and discharge

Ophthalmology was consulted in the inpatient setting immediately after deficits were noted. Ophthalmology recommended carboxymethylcellulose gel tears and a moisture chamber with Tegaderm due to exposure keratopathy from left facial weakness. Left eye abduction was noted to be weak, and patching was recommended to combat diplopia. The patient was discharged with a referral to outpatient physical therapy. She was also instructed to follow up with ophthalmology.

## Diagnostic assessment

The patient's clinical findings and neuroimaging suggest a left dorsal pons localization because of the missed VIM target. The fascicle of the left facial nerve loops around the ipsilateral abducens nucleus, thus an ipsilateral gaze palsy and facial nerve palsy are highly indicative of a lesion in this area.

## Follow-up and outcomes

### Neuro-ophthalmology evaluation

At the initial neuro-ophthalmology evaluation 2 weeks after her aborted DBS surgery, the patient was still having left facial weakness and problems closing her left eye completely. She reported double vision, particularly with a horizontal gaze. Motility testing revealed −3 adduction of the right eye and −3 abduction of the left eye consistent with left gaze palsy. There was an 18 prism diopter alternating esotropia by alternate cover testing at distance without correction. This esotropia was 16 prism diopters in the right gaze, but difficult to reliably measure in the left gaze due to the gaze palsy. Saccades, smooth pursuit movements, and the vestibulo-ocular reflex were all impaired. Left upper and lower facial weakness was noticeable with intact sensation. The patient reported some difficulty speaking and swallowing, but no deficits were clearly localized to the midbrain. Her best corrected visual acuity and confrontation visual fields were normal. Pupils were round, equal, and reactive to light with no afferent pupillary defect. A 2 mm lagophthalmos on the right eye was noted. Upon slit lamp examination, the left cornea displayed 1.5+ diffuse superficial punctate keratitis. No eye misalignment was noticeable when standing near the patient, but esotropia was evident at a distance.

### Neuro-ophthalmology follow-up #1

One month after the initial consultation, the patient said that her vision was about the same, endorsing continued horizontal double vision that worsened with distance. She had started vision therapy and noticed that taping over her superior lens somewhat helped with the double vision. Motility testing revealed −2.5 adduction of the right eye and −2.5 abduction of the left eye consistent with mild improvement. She continued to have an 18 prism diopter esotropia at distance in primary gaze. Left upper and lower facial weakness was still evident, though the patient subjectively felt like her facial strength was improving.

### Neuro-ophthalmology follow-up #2

Almost 8 months after surgery, the patient reported that she was still having double vision. However, this was improved when she wore her glasses with 10 prism diopters of the base out fresnel prism on the left lens. Her corrected visual acuity was OD 20/20 and OS was 20/60 +2. Visual acuity in the left eye was felt to be decreased due to the fresnel prism. Motility testing revealed −1.5 adduction of the right eye and −1.5 abduction of the left eye, consistent with further improvement of the left gaze palsy. Left-sided facial strength had improved.

### Neuro-ophthalmology follow-up #3

Almost 10 months after the initial VIM DBS surgery, the patient had strabismus surgery involving a recession of bilateral medial rectus muscles, which led to the elimination of esotropia. Her diplopia improved by 50–60% after surgery. She still had intermittent diplopia and small alternating vertical deviation upon lateral gaze in either direction. Motility testing showed −1 adduction on the right eye and −1 abduction of the left eye.

## Discussion

Deep brain stimulation (DBS) is a procedure that was first approved in the 1990's and has had high success with improving symptomatic outcomes in patients with disabling motor disorders (7). Through neurosurgery, electrodes are placed near certain structures in the brain and then connected to a pulse generator that is implanted into the chest. Though its exact mechanism of action is unknown, different models and hypotheses have been explored to understand its pathophysiology. The “disruption hypothesis” suggests that DBS interrupts inappropriate signaling to nuclei, which in turn disrupts abnormal electrical flow through the corticobasal ganglia loop in pathologic conditions (8). Furthermore, it is believed that this disruption of electrical activity modulates neuroplasticity and plays a role in neurogenesis (9).

Among conditions that utilize DBS for intervention, severe or worsened adverse effects are seen among patients with Parkinson's Disease (PD) (6), with up to 50% of patients potentially experiencing adverse events related to the procedure. In subthalamic nucleus (STN) DBS, mental status/behavioral changes are the most common procedure-related adverse events, with confusion topping the list. For globus pallidus internus (GPi) DBS, mental status/behavioral changes are also the highest reported followed by speech disturbances (10). Complications can be further derived from stimulation, such as electrical flow expanding through association regions and causing non-motor symptoms including laughter and hypomania (11).

In essential tremors, the classic target for DBS placement is the ventral intermediate nucleus of the thalamus (VIM), a structure involved in the dentato-rubro-thalamic tract and important for the abnormal electrical conduction in essential tremors. Other comparable targets in DBS for essential tremors include the posterior thalamic area (PSA) and Zona incerta (Zi) (12). The DBS procedure is efficacious in reducing tremor severity, particularly up to 10 years after the surgery, with efficacy decreasing after 10 years (13). The most common side effects from DBS placement include gait ataxia and dysarthria, reported among all three anatomic targets. In this report, we described a patient with left gaze and facial nerve palsies as the primary complications following a missed VIM target during DBS neurosurgery for refractory essential tremors.

Isolated horizontal gaze palsy is caused by interruption of the abducens nucleus or paramedian pontine reticular formation (PPRF). Inciting events include infarction, inflammation, hemorrhage, or metastasis, with gaze palsy sometimes seen as part of multiple sclerosis or neuromyelitis optica spectrum disorders (14). Clinical ophthalmologic presentations can include impairment of smooth pursuit and saccadic eye movements and can be accompanied by horizontal or vertical nystagmus. In addition to her gaze palsy, this patient also suffered from diplopia due to esotropia. Esotropia causing diplopia has been reported in association with horizontal gaze palsies due to dorsal pontine lesions (15, 16). For isolated facial nerve palsies, almost 75% of causes are attributed to Bell's palsy (17). Other less common causes of facial palsies include cerebellopontine angle tumors and pontine infarcts (18).

For the patient presented in this case, isolated left gaze and facial nerve palsies were attributed to canal insertion into the left dorsal pons during a VIM DBS thalamotomy. The abducens (sixth) and facial (seventh) cranial nerves reside in the pons. The abducens nucleus innervates the ipsilateral lateral rectus muscle and the contralateral medial rectus subnucleus of the oculomotor nuclear complex by way of the MLF. During surgery, the target of interest was the ventral intermediate nucleus of the thalamus, a common landmark for DBS electrode placement in refractory clinical cases. Anatomically, the thalamus is located superomedial to the pons. Overshooting of catheter insertion during initial entry caused an interruption of the pontine pathway. Clinically, this presented as weakness of left horizontal gaze and left facial weakness. Gaze palsies are typically due to interruption of the ipsilateral PPRF, ipsilateral abducens nucleus, or both. PPRF lesions tend to cause primarily saccadic gaze palsies, and as such, it is most likely that in our patient the abducens nucleus was affected (19).

## Conclusion

Our case report demonstrates that VIM DBS can present as isolated left horizontal gaze and facial nerve palsies if the thalamic target is overshot during surgery. While DBS can provide acute improvements in refractory movement disorders, neurosurgeons and neuro-ophthalmologists should be wary of the anatomic proximity between the thalamus and the important structures of the brainstem when understanding potential complications of this procedure. Surgery risks should further consider the holistic effects of visual impairment, taking into account the patient's baseline visual function. All patients who undergo VIM DBS surgery should be assessed for potential cranial nerve deficits post-placement.

## Patient perspective

It should be noted that for the patient presented in this report, visual impairment as a consequence of surgery led to many significant changes, including not being able to drive, not being able to care for others, and a loss of independence. This took a toll on the patient's mental health, ultimately leading to a dosage increase of her Lexapro. The mental health effects of impaired visual function should be considered when weighing the risks and benefits of surgery. For this patient, there was an original decrease in tremor severity right after surgery, but her tremor has since returned to baseline prior to neurosurgery.

## Data availability statement

The original contributions presented in the study are included in the article/supplementary material, further inquiries can be directed to the corresponding author.

## Ethics statement

Written informed consent was obtained from the individual(s) for the publication of any potentially identifiable images or data included in this article.

## Author contributions

SG examined the patient, established the diagnosis, obtained the images, and revised the manuscript. VC reviewed the literature and wrote the whole manuscript. All authors listed have made a substantial, direct, and intellectual contribution to the work and approved it for publication.

## References

[1] Lozano AM Lipsman N Bergman H Brown P Chabardes S Chang JW. Deep brain stimulation: current challenges and future directions. Nat Rev Neurol. (2019) 15:148–60. 10.1038/s41582-018-0128-2 30683913 PMC6397644

[2] Koeglsperger T Palleis C Hell F Mehrkens JH Bötzel K. Deep Brain Stimulation Programming for Movement Disorders: Current Concepts and Evidence-Based Strategies. Front Neurol. (2019) 10:410. 10.3389/fneur.2019.00410 31231293 PMC6558426

[3] Rajput AH Rajput A. Medical Treatment of Essential Tremor. J Cent Nerv Syst Dis. (2014) 6:13570. 10.4137/jcnsd.s13570 24812533 PMC3999812

[4] Nazzaro JM Lyons KE Pahwa R. Brain Stimulation: Chapter 13. In: Deep Brain Stimulation for Essential Tremor. Elsevier Inc. (2013). 10.1016/B978-0-444-53497-2.00013-9 24112892

[5] Kim MJ Chang KW Park SH Chang WS Jung HH Chang JW. Stimulation-induced side effects of deep brain stimulation in the ventralis intermedius and posterior subthalamic area for essential tremor. Front Neurol. (2021) 12:678592. 10.3389/fneur.2021.678592 34177784 PMC8220085

[6] Buhmann C Huckhagel T Engel K Gulberti A Hidding U Poetter-Nerger M. Adverse events in deep brain stimulation: A retrospective long-term analysis of neurological, psychiatric and other occurrences. PLoS ONE. (2017) 12:e0178984. 10.1371/journal.pone.0178984 28678830 PMC5497949

[7] Fariba KA Gupta V. Deep Brain Stimulation. Treasure Island, FL: StatPearls Publishing. (2022). 32496727

[8] Chiken S Nambu A. Mechanism of deep brain stimulation: inhibition, excitation, or disruption? Neuroscientist. (2016) 22:313–22. 10.1177/1073858415581986 25888630 PMC4871171

[9] Herrington TM Cheng JJ Eskandar EN. Mechanisms of deep brain stimulation. J Neurophysiol. (2016) 115:19–38. 10.1152/jn.00281.2015 26510756 PMC4760496

[10] Videnovic A Metman LV. Deep brain stimulation for Parkinson's disease: prevalence of adverse events and need for standardized reporting. Mov Disord. (2008) 23:343–9. 10.1002/mds.21753 17987644

[11] Alonso-Frech F Fernandez-Garcia C Gómez-Mayordomo V. Non-motor adverse effects avoided by directional stimulation in Parkinson's disease: A case report. Front Neurol. (2021) 12:786166. 10.3389/fneur.2021.786166 35173666 PMC8843015

[12] Kübler D Kroneberg D Al-Fatly B Schneider G-H Ewert S van Riesen C. Determining an efficient deep brain stimulation target in essential tremor - Cohort study and review of the literature. Parkinsonism Relat Disord. (2021) 89:54–62. 10.1016/j.parkreldis.2021.06.019 34225135

[13] Paschen S Forstenpointner J Becktepe J Heinzel S Hellriegel H Witt K. Long-term efficacy of deep brain stimulation for essential tremor: An observer-blinded study. Neurology. (2019) 92:e1378–86. 10.1212/WNL.0000000000007134 30787161

[14] Ewe R White OB Burke A. Isolated horizontal gaze palsy: observations and explanations. Front Neurol. (2017) 8:611. 10.3389/fneur.2017.00611 29187832 PMC5694745

[15] Coats DK Avilla CW Lee AG Paysse EA. Etiology and surgical management of horizontal pontine gaze palsy with ipsilateral esotropia. J AAPOS. (1998) 2:293–7. 10.1016/S1091-8531(98)90086-6 10646751

[16] Somer D Cinar FG Kaderli A Ornek F. Surgical planning and innervation in pontine gaze palsy with ipsilateral esotropia. J Am Assoc Pediatr Ophthalmol Strabis. (2016) 20:410–4. 10.1016/j.jaapos.2016.07.222 27651233

[17] Holland NJ Julian Holland N Weiner GM. Recent developments in Bell's palsy. BMJ. (2004) 329:553–7. 10.1136/bmj.329.7465.553 15345630 PMC516110

[18] Agarwal R Manandhar L Saluja P Grandhi B. Pontine stroke presenting as isolated facial nerve palsy mimicking Bell's palsy: a case report. J Med Case Rep. (2011) 5:1–4. 10.1186/1752-1947-5-287 21729278 PMC3141723

[19] Kochar PS Kumar Y Sharma P Kumar V Gupta N Goyal P. Isolated medial longitudinal fasciculus syndrome: Review of imaging, anatomy, pathophysiology and differential diagnosis. Neuroradiol J. (2018) 31:95–9. 10.1177/1971400917700671 28541157 PMC5789990

